# Agrobiodiversity and in situ conservation in ethnic minority communities of Xishuangbanna in Yunnan Province, Southwest China

**DOI:** 10.1186/s13002-017-0158-7

**Published:** 2017-05-15

**Authors:** Shicai Shen, Gaofeng Xu, Diyu Li, David Roy Clements, Fudou Zhang, Guimei Jin, Jianyong Wu, Pingfang Wei, Song Lin, Dayuan Xue

**Affiliations:** 1 Agricultural Environment and Resource Research Institute, Yunnan Academy of Agricultural Sciences, Kunming, 650205 China; 2 Biology Department, Trinity Western University, 7600 Glover Road, Langley, BC V2Y 1Y1 Canada; 3 Nanjing Institute of Environmental Sciences, Ministry of Environmental Protection, Nanjing, 210042 China; 4 Economical Crop Station of Jinghong City in Xishuangbanna, Jinghong, 666100 China; 5 Agricultural Products Quality and Safety Inspection Station of Menghai County in Xishuangbanna, Menghai, 666200 China; 6 College of Life and Environmental Sciences, Minzu University of China, Beijing, 100081 China

**Keywords:** Xishuangbanna, Agrobiodiversity, Diversity change, In situ conservation, Ethnic minority

## Abstract

**Background:**

Xishuangbanna of Yunnan Province, southwest of China belongs to a global biodiversity and cultural hotspot. Agrobiodiversity plays an essential role in local livelihoods and traditional culture in the region. However, preliminary studies suggest that diversity of crop plants and livestock species is declining. We hypothesized that agrobiodiversity and traditional means of preserving agrobiodiversity are threatened because of changes in government policy in favor of commercial plantations, land use change and changes in traditional agricultural practices. We investigated whether or not agrobiodiversity was declining, the specific causes, and signs of active biodiversity conservation practices in ethnic minority communities of Xishuangbanna which could form the basis for extensive in situ conservation programmes.

**Methods:**

A series of field studies to document trends in agrobiodiversity were conducted in different ethnic minority communities in Menghai County, Mengla County and Jinghong City of Xishuangbanna of Yunnan Province, southwest of China between July 2015 and February 2016. Data was obtained through the use of semi-structured questionnaires, field observation and participatory rural appraisal (PRA) tools. A total of 360 ethnic households provided information on current status, functions, characteristics, changes, and threatened factors of farming crop and livestock resources. Some measures for in situ conservation of agricultural biological resources were also researched using PRA methods.

**Results:**

Two hundred twenty-six crop varieties belonging to 31 families, 71 genera and 101 species were identified in Xishuangbanna, which included 83 vegetable crops, 77 food crops, 24 spice crops, 22 fruit crops, 13 cash crops, 6 oil crops, and 1 cloth crop, respectively. There were 15 livestock varieties, belonging to 6 major species: cattle, pigs, goats, chickens, ducks, and geese. Different crop and livestock resources had their own characteristics, functions and threatened factors. Since 2002, agroecosystem, crop diversity and livestock diversity have declined greatly over the Xishuangbanna region as a whole under implementation of the Sloping Land Conversion Program (SLCP). Swidden agriculture was completely eliminated under this program and gradually replaced by large land areas devoted to rubber, tea and banana plantations. Villager numbers engaging in farming production and population of crops and livestock were greatly decreased, particularly in terms of production of local traditional varieties. However, some in situ conservation measures such as seeds preservation, planting of traditional crops and raising livestock have played an important role in local agrobiodiversity conservation.

**Conclusion:**

Abundant agricultural resources and agrobiodiversity are critical to the local livelihood and maintenance of traditional culture in Xishuangbanna. However, agrobiodiversity and related traditional culture have been greatly impacted by implementation of the SLCP since 2002. Therefore, in future conservation of agrobiodiversity, incorporating some sustainable protection measures based in local communities such as convening seed exchange fairs, conserving traditional varieties in permanent plots, making a visual documentary of indigenous cultivation, and providing traditional agricultural products to tourists should be carefully considered and adopted.

## Background

Biodiversity is the material foundation of human survival and development and also is an important symbol to measure the environmental quality status and degree of ecological civilization in a region or a country. Biodiversity refers to the sum total of different animals, plants and organisms living on Earth and includes species and genetic diversity, as well as the variety of habitats and ecosystems where they live [1, 2]. Biodiversity functions to provide direct and beneficial products to human, regulation of climate and environment, formation of unique cultures, and other functions [3].

Agrobiodiversity plays a significant part in global biodiversity system [4]. The maintenance of agrobiodiversity is extremely important for production of food and other agricultural products, as well as for human development, including food security, nutrition and means for improving livelihood. Agrobiodiversity is the general term for all organisms related to food and agricultural production and can be demonstrated to be an important means of meeting human health and development goals [5, 6]. Promotion of agrobiodiversity involves utilization of species, germplasm conservation, utilization of resource management, and agricultural ecological environment protection. Farmers and agricultural producers are the main managers of agrobiodiversity and have abundant knowledge of biodiversity management and maintenance. Agriculture contributes to conservation and sustainable use of biodiversity, but it is also an important driving force for loss of biodiversity [6, 7]. However, currently agrobiodiversity and related traditional knowledge are often neglected and easily lost because of biological invasion, the advent of new crop varieties and the application of technology related to the production of transgenic organisms.

Xishuangbanna is a multinational region, which harbors much of the cultural diversity and biodiversity of China [8]. Swidden agriculture is central to local livelihood, agrobiodiversity and traditional culture in Xishuangbanna [7]. Widely known as the “Animal and Plant Kingdom”, Xishuangbanna is rich in animal and plant species resources. There are over 4669 vascular plant species, 700 species of higher animals and 1500 species of insects [8]. The number of these species makes up 21%, 40.3% and 15% of the total of Yunnan, or 13% 24% and 6% of that of China, respectively [8–10]. Moreover, there were more than 577 cultivated plant species in Xishuangbanna before 2011 [11]. However, in recent years, with rapid economic development and changing national policy, agricultural ecosystems and agrobiodiversity in the region face tremendous challenges.

We hypothesized that agrobiodiversity and traditional means of preserving agrobiodiversity are threatened by changes in government policies, land use change and abandonment of traditional agricultural practices. Therefore, our objective was to investigate whether there were indications that agrobiodiversity was in decline, and how much it has declined. Furthermore, we examined the role of in situ conservation via ethnic minorities in Menghai County, Mengla County and Jinghong City in Xishuangbanna from July 2015 to February 2016. Through a survey we sought to investigate what measures might provide for conservation of local biodiversity and traditional culture in Xishuangbanna.

## Methods

### Study area

Xishuangbanna Dai Autonomous Prefecture is situated in the south end of Yunnan Province, southwest China, between 99° 56’-101° 50’E and 21° 08’-22° 36’N. To the west and east, it borders Jiangcheng County and Puer City, and to the northwest, it shares a border with Lancang County in Yunnan Province, and shares a boundary of 1069 km with Myanmar and Laos in the southeast, south and southwest. The total area is 19, 223 km^2^ of which 95% is mountainous area. The elevation ranges from 477-2429 m, and annual average temperature is around 15-22 °C with an annual rainfall of 1138.6-2431.5 mm [7]. The region is inhabited by 13 ethnic groups, dominated by Dai people, supplemented by Han, Hani, Yi, Lahu, Bulang, Jinuo, Yao, Miao, Bai, Hui, Wa, and Zhuang people. Due to special geographical location, complicated terrain and superior climates, there are abundant agricultural biological resources and traditional culture [7, 12–14].

### Methods

The main aims of the study were to investigate indicators of agrobiodiversity decline and to examine the role of in situ conservation via ethnic minorities in Xishuangbanna. According to elevation and minority differences, a total of 24 ethnic minority villages of 11 townships in Menghai County, Mengla County and Jinghong City of Xishuangbanna were selected. The elevation range was 538-1660 m and ethnic groups included Dai, Hali, Lahu, Yao, Yi, Jinuo, Bulang, Miao, and Han.

Based on the selected villages, field data collection was undertaken using questionnaires, interviews, group discussions, key informants, observations, and participatory rural appraisal/rapid rural appraisal (PRA/RRA) from July 2015 to February 2016. Information gathered included: village name, ethnic groups, population, farming calendar, land area, and livestock. General information collected at the household level, included social and economic situation, the respondent’s basic situation and agricultural practices were supported by a survey using a questionnaire sampling 360 households during a field survey. This relatively large sample size enabled us to assess common trends despite some differences in terminology among respondents, taking care to account for nomenclature differences between different localities. Household information included: crop cultivation (crop type, origin, grow area, yield, function, purpose, problems, and conservation means) and animal production (herd size, function, purpose, problems, and conservation methods). Participatory Rural Appraisal (PRA) [15, 16] and ethnobotanical and anthropological methods [17, 18] were used to facilitate participation of local villagers in extraction and analysis of agrobiodiversity and related traditional agricultural knowledge in the field. Interviews were conducted individually and collectively, taking into consideration gender and age differences. Household samples were selected at random in 24 ethnic villages of 360 households in Menghai County, Mengla County and Jinghong City.

### Data analysis

All primary data and sources were captured and analyzed with qualitative and quantitative methods and tools. In some cases like agrobiodiversity trends, qualitative comparisons were more appropriate than quantitative comparisons. Field data was entered in Microsoft Excel and analysed with the Statistical Package for Social Scientist (SPSS). Quantitative data was analyzed by using descriptive statistics through calculating frequency, percentage, means, and tables to present the outputs.

## Results

### Community farming calendar

Agricultural production was the most important production activity in rural communities of Xishuangbanna, and each year villagers spent a lot of time on cultivation, management and harvest of food crops and cash crops (Table 1). Due to high temperatures and adequate rainfall, crops could be grown over 2-3 seasons per year. Vegetables especially could be cultivated and harvested at any time because of faster growth rates and shorter growth periods. The cultivation time of crops for local villagers was usually influenced by market demand and cash crop types. Rubber tree tapping (March to November) and tea collection (February to October) were the main farming activities. Corn and vegetables could be cultivated at any time throughout the year, but winter corn and vegetables were mainly sown in January. Upland rice was generally sown in March, paddy rice was sown from May to June, and fall corn was sown from April to May (Table 1).

**Table 1:** Community farming calendar in Xishuangbanna

Month	Farming activities and traditional customs activities
January	Winter corn management, weeding and fertilizing, sweet corn harvest, vegetable and peanut sowing, house building, wedding, tea weeding but not fertilizing, sugarcane harvest, *Amomum villosum* weeding and old branch and leaf cutting
February	Spring tea collection, fresh corn harvest, rubber tree weeding and fertilizing, sugarcane harvest, *Amomum villosum* weeding and old branch and leaf cutting
March	Tea collection, rubber tree tapping, upland rice sowing and weeding, sugarcane harvest
April	Tea collection, corn sowing, rubber tree tapping, winter corn harvest, sugarcane harvest
May	Tea collection, corn sowing, rubber tree tapping, winter corn harvest, paddy rice seedling and transplanting, upland rice harvest.
June	Tea collection, rubber tree tapping, paddy rice seedling and transplanting, upland rice harvest
July	Tea collection, rubber tree tapping, wild mushroom collection
August	Tea collection, rubber tree tapping, wild mushroom collection, *Amomum villosum* collection
September	Tea collection, corn harvest, rubber tree tapping, paddy rice collection
October	Tea collection, rubber tree tapping, paddy rice collection, vegetable sowing, sweet corn sowing, sugarcane leaf cutting
November	Winter corn harvest, wedding, fuel wood collection, house building, sugarcane leaf cutting
December	Winter corn harvest, wedding, fuel wood collection, house building, sugarcane harvest, vegetable and potato sowing

Livestock was also an important farming activity. All households raised large and small livestock (cattle, goats, pigs, chickens, ducks, and geese), and family members spent considerable time on animal husbandry. There were no special grasslands in Xishuangbanna, so cattle and goats were mainly herded to wastelands and mountains jointly owned by several households but pigs were pen-raised. Poultry such as chickens, ducks and geese were freely grazed near villagers’ houses.

### Agrobiodiversity

#### Crop genetic resources and functions

There were 226 crop varieties belonging to 31 families, 71 genera and 101 species grown in Xishuangbanna (Table 2). The paddy rice (23 varieties) and corn (23 varieties) had the greatest number of varieties, followed by upland rice (21 varieties). There were 165 local crop varieties and 61 imported crop varieties, accounting for 73% and 27% of all crop varieties grown, respectively. Of the 226 crop varieties grown, there were 83 vegetable crops, 77 food crops, 24 spice crops, 22 fruit crops, 13 cash crops, 6 oil crops, and 1 cloth crop (Table 2).

**Table 2:** Summary of type and characteristics of crops in Xishuangbanna

Family	Scientific name	Local variety	Imported variety	Resource type	Cultivation status	Main characteristics and uses
Amaranthaceae	*Amaranthus tricolor*	2	0	Vegetable	Some households and little land area per household	Annual, green or red color, human consumption
	*Spinacia oleracea*	1	0	Vegetable	Some households and little land area per household	Annual, human consumption, sale
Amaryllidaceae	*Allium fistulosum*	1	1	Spice	For local variety, many households and little land area per household For imported variety, few households and little land area per household	For local variety, small root, short length, spicy, more fragrant, human consumption, sale For imported variety, large length, big root, human consumption
	*Allium sativum*	1	1	Spice	For local variety, many households and little land area per household For imported variety, few households and little land area per household	For local variety, small root, short length, spicy, more fragrant, human consumption, sale For imported variety, large length, big root, human consumption
	*Allium tuberosum*	1	0	Spice	A lot of households and little land area per household	Tall and thin plant, human consumption, prickles
Anacardiaceae	*Mangifera indica*	2	0	Fruit	Many households and little land area per household	Perennial, good tasty, more fragrant, human consumption, sale
Apiaceae	*Apium graveolens*	2	0	Vegetable	A lot of households and middle land area per household	Good tasty, human consumption, sale
	*Coriandrum sativum*	1	0	Spice	A lot of households and little land area per household	Good tasty, human consumption, oil making
	*Daucus carota* subsp. *sativus*	1	0	Vegetable	Few households and little land area per household	Short root, human consumption, sale
	*Eryngium foetidum*	1	0	Spice	Some households and little land area per household	Human consumption
	*Foeniculum vulgare*	1	1	Vegetable	For local variety, some households and little land area per household For imported variety, some households and little land area per household	For local variety, grow fast, good fragrant, human consumption, seeds for medicine For imported variety, grow slow, human consumption, seeds for medicine
Araceae	*Amorphophallus albus*	1	0	Vegetable	Some households and little land area per household	Perennial, small root, human consumption, sale
	*Amorphophallus krausei*	1	0	Vegetable	Some households and little land area per household	Perennial, small root, human consumption, sale
	*Amorphophallus yuloensis*	1	0	Vegetable	Some households and little land area per household	Perennial, small root, human consumption, sale
	*Amorphophallus yunnanensi*s	1	0	Vegetable	Some households and little land area per household	Perennial, small root, human consumption, sale
	*Colocasia esculenta*	1	0	Vegetable	Some households and little land area per household	Perennial, small root, human consumption, fodder, sale
	*Colocasia gigantea*	1	0	Vegetable	Some households and little land area per household	Perennial, big root, human consumption, fodder
Asteraceae	*Glebionis coronaria*	1	0	Vegetable	Some households and little land area per household	High yield, human consumption
	*Helianthus annuu*	3	0	Oil	Some households or no households and middle land area per household	Low yield, drought resistance, barren resistance, snacks, making oil
	*Lactuca sativa*	0	1	Vegetable	Many households and little land area per household	Perennial, high yield, human consumption, fodder, sale
Brassicaceae	*Brassica juncea*	3	0	Vegetable	Many households and little land area per household	Low yield, good tasty, fodder, prickles, human consumption
	*Brassica oleracea* var. *capitata*	1	1	Vegetable	For local variety, a lot of households and little land area per household For imported variety, few households and little land area per household	For local variety, low yield, more sweet, prickles, human consumption For imported variety, high yield, human consumption, fodder
	*Brassica rapa* subsp. *chinensis*	1	1	Vegetable	For local variety, a lot of households and little land area per household For imported variety, a lot of households and little land area per household	For local variety, low yield, good tasty, small length and leaf, human consumption For imported variety, high yield, high length and big leaf, human consumption, fodder
	*Brassica rapa* subsp. *pekinensis*	2	0	Vegetable	Many households and large land area per household	High yield, human consumption, fodder, sale
	*Raphanus sativus*	1	1	Vegetable	For local variety, few households and little land area per household For imported variety, a lot of households and little land area per household	For local variety, low yield, more sweet, human consumption, fodder, making kimchi For imported variety, high yield, human consumption, fodder, making kimchi
Bromeliaceae	*Ananas comosus*	1	0	Fruit	Few households and little land area per household	Good tasty, sweet, human consumption, sale
Cannabaceae	*Cannabis sativa*	0	1	Cash	Some households and large land area per household	Extension by government since 2004, 2-3 m height, sale
Caricaceae	*Carica papaya*	1	0	Fruit	Many households and little land area per household	Perennial, sweet, human consumption, sale
Convolvulaceae	*Ipomoea aquatica*	1	1	Vegetable	For local variety, some households and little land area per household For imported variety, few households and large land area per household	For local variety, low yield, more tasty, human consumption, fodder For imported variety, high yield, mainly for sale, fodder, human consumption
	*Ipomoea batatas*	2	1	Vegetable	For local variety, some households and little land area per household For imported variety, some households and large land area per household	For local variety, low and middle yield, more tasty, fodder, human consumption, sale For imported variety, high yield, fodder, human consumption, sale
Cucurbitaceae	*Benincasa hispida*	2	0	Vegetable	For local variety, a lot of households and large area per household For imported variety, few households and little land area per household	For local variety, middle yield, more fragrant, 2-3 kg /one, human consumption, sale For imported variety, high yield, 10-20 kg /one, fodder, sale
	*Citrullus lanatus*	0	1	Fruit	Few households and large land area per household	High yield, sale, human consumption
	*Cucumis hystrix*	1	0	Vegetable	few households and little land area per household	Prostrating, high yield, more sweet, human consumption, sale
	*Cucumis melo*	1	0	Vegetable	few households and little land area per household	Climbing, middle yield, more sweet, human consumption, sale
	*Cucumis sativus*	2	1	Vegetable	For local variety, some households and little land area per household For imported variety, a few households and little land area per household	For local variety, no thorn, short, prostrating or climbing, low or high yield, more sweet, human consumption, sale For imported variety, thorn, long, climbing, high yield, human consumption, sale
	*Cucurbita moschata*	4	1	Vegetable	For local variety, some households or no households and little land area per household, or few households and large land area per household For imported variety, many households and large land area per household	For local variety, different shape, low yield, more sweet, fodder, human consumption, sale For imported variety, high yield, fodder, human consumption, sale
	*Lagenaria siceraria*	3	0	Vegetable	Some households and little land area per household or few households and large land area per household	Different yield, making cucurbit flute, human consumption, sale, fodder, making bailer
	*Luffa aegyptiaca*	2	0	Vegetable	A lot of households and little land area per household	Middle and high yield, good tasty, human consumption, washing dishes
	*Momordica charantia*	1	1	Vegetable	For local variety, few households and little land area per household For imported variety, few household and large land area per household	For local variety, low yield, more bitter, human consumption, sale For imported variety, high yield, less bitter, sale, human consumption
	*Sechium edule*	1	0	Vegetable	Many households and little land area per household	Climbing, disease resistance, better tasty and more pretty fruit in winter, human consumption, sale
Dioscoreaceae	*Dioscorea esculenta*	2	0	Vegetable	Some households and little land area per household	Perennial, low yield, small and middle root, human consumption
	*Dioscorea opposit*a	2	0	Vegetable	Some households and little land area per household	Perennial, low yield, small and middle root, human consumption
Euphorbiaceae	*Hevea brasiliensis*	0	1	Cash	A lot of households and large land area per household	Perennial, sale
	*Manihot esculenta*	0	1	Cash	Few households and large land area per household	Perennial, sale, human consumption
Fabaceae	*Arachis hypogaea*	2	1	Oil	For local variety, some households and little land area per household For imported variety, few households and little land area per household	For local variety, white and yellow seeds, low yield, human consumption, sale, making oil For imported variety, color seeds, high yield, human consumption, sale, making oil
	*Glycine max*	2	0	Vegetable	Some households and little land area per household	Low yield, yellow and white seed, bean curd, bean milk, bean sprout, good tasty, more fragrant, human consumption
	*Lablab purpureu*	2	0	Vegetable	Some households and no households and little land area per household	Perennial, human consumption
	*Phaseolus coccineus*	2	0	Vegetable	Some households and little land area per household	High yield, good tasty, human consumption
	*Phaseolus vulgaris*	3	0	Vegetable	Some households and no households and little land area per household	Low yield, good tasty, barren resistance, human consumption, sale
	*Pisum sativum*	1	1	Vegetable	For local variety, few households and little land area per household For imported variety, many households and little land area per household	For local variety, hard shuck, human consumption For imported variety, soften shuck, human consumption
	*Psophocarpus tetragonolobus*	2	0	Vegetable	Some households and little land area per household	Perennial, high yield, good tasty, disease resistance, human consumption
	*Senegalia pennata*	1	0	Vegetable	Few households and little land area per household	Perennial, low yield, human consumption
	*Vicia faba*	1	0	Vegetable	Some households and little land area per household	Low yield, good tasty, more fragrant, human consumption
	*Vigna angularis*	1	0	Vegetable	Few households and little land area per household	Low yield, climbing, porridge, cake making, human consumption
	*Vigna radiata*	1	0	Vegetable	Some households and little land area per household	Low yield, good tasty, disease resistance, human consumption
	*Vigna sinensis*	0	1	Vegetable	Few households and little land area per household	High yield, human consumption, sale
	*Vigna umbellata*	1	0	Vegetable	Some households and little land area per household	Low yield, porridge, human consumption
	*Vigna unguiculata*	0	2	Vegetable	Some households and large land area per household	High yield, 3-4 times collection, human consumption, sale
Lamiaceae	*Agastache rugosa*	1	0	Spice	Some households and little land area per household	Middle yield, more fragrant, human consumption
	*Elsholtzia kachinensis*	1	0	Spice	A lot of households and little land area per household	Human consumption, sale
	*Mentha haplocalyx*	1	0	Spice	A lot of households and little land area per household	Human consumption, sale
	*Ocimum basilicum*	2	0	Spice	A lot of households and little land area per household	Good tasty, good smell, human consumption, sale
	*Perilla frutescens*	2	0	Spice	Few households and little land area per household	Low yield, more fragrant, human consumption
	*Pogostemon cablin*	1	0	Spice	Some households and little land area per household	Low yield, more fragrant, human consumption
Moraceae	*Artocarpus heterophyllus*	1	0	Fruit	A lot of households and little land area per household	Perennial, good smell, human consumption, sale
Musaceae	*Musa acuminata*	2	0	Fruit	For local variety, many households and little land area per household For imported variety, many households and large land area per household	For local variety, little fruit, low yield, human consumption, fodder, sale For imported variety, large fruit, high yield, human consumption, fodder, sale
	*Musa basjoo*	1	0	Fruit	A lot of households and little land area per household	Leaf for multi-purposes, human consumption, fodder, sale
Myrtaceae	*Psidium guajava*	1	0	Fruit	Many households and little land area per household	Perennial, sweet, human consumption, sale
Passifloraceae	*Passiflora caerulea*	2	0	Fruit	Many households and little land area per household	Perennial, good smell, human consumption
Pedaliaceae	*Sesamum indicum*	2	0	Spice	Few households or no households and little land area per household	Good tasty, making oil, human consumption
Poaceae	*Coix lachryma-jobi*	2	0	Food	Few households or not household and large land area per household	Middle and high yield, good tasty, good smell, suitable for high altitude, human consumption, making wine, fodder, sale
	*Cymbopogon citratus*	1	0	Spice	A lot of households and little land area per household	Perennial, spice for chicken and pork cooking
	*Oryza sativa*	7	16	Food	For local variety, a few households and little land area per household For imported variety, a lot of households and large land area per household	For local variety, good tasty, good smell, low yield, suitable for high altitude, human consumption, disease resistance, barren tolerance, sale For imported variety, suitable for low and middle altitude, high yield, human consumption, sale
	*Oryza sativa* var. *spontanea*	21	0	Food	A few households and little land area or large land area per household	Good tasty, good smell, low yield, suitable for high altitude, human consumption, disease resistance, barren tolerance
	*Saccharum officinarum*	0	2	Cash	A lot of households and large land area per household	Imported since 1992, making sugar, sale, few human consumption
	*Sorghum bicolor*	3	0	Food	Few households or not household and large land area per household	Middle yield, good tasty, good smell, fodder, human consumption, making wine, making broom, washing dishes, dying cloth
	*Triticum aestivum*	1	2	Food	For local variety, no households per household For imported variety, few households no household and large land area per household	For local variety, low yield, good tasty, human consumption, making wine For imported variety, high yield, less tasty, just for fodder
	*Zea mays*	10	13	Food	For local variety, a few households or no households and little land area per household For imported variety, a lot of households or few households and large land area per household	For local variety, good tasty, low yield, suitable for high altitude, barren resistance, disease resistance, fodder, making wine, human consumption, sale For imported variety, suitable for low and middle altitude, high yield, fodder, making wine, human consumption, sale
Polygonaceae	*Fagopyrum tataricum*	2	0	Food	Few households and little land area per household	Low yield, barrel resistance, making wine, human consumption, fodder
Ranunculaceae	*Anemone vitifolia*	1	0	Cloth	No households	Middle yield, spinning, weaving, spinning cloth, seeds for medicine
Rhamnaceae	*Ziziphus mauritiana*	0	1	Fruit	Few households and large land area per household	Middle yield, sale, human consumption
Rosaceae	*Prunus persica*	2	0	Fruit	Few households and little land area per household	Perennial, good tasty, sweet, human consumption, sale
	*Prunus salicina*	1	0	Fruit	Some households and little land area per household	Perennial, good tasty, sweet, human consumption, sale
Rubiaceae	*Coffea arabica*	0	1	Cash	Some households and large land area per household	Perennial, low yield, more fragrant, sale
	*Coffea liberica*	0	1	Cash	Some households and large land area per household	Perennial, middle yield, sale
Rutaceae	*Citrus grandis*	1	0	Fruit	Some households and little land area per household	Perennial, good tasty, sweet, human consumption, sale
	*Citrus limon*	2	0	Fruit	Some households and little land area per household	Perennial, good tasty, sweet, human consumption, sale
	*Citrus medica*	1	0	Fruit	Some households and little land area per household	Perennial, more fragrant, human consumption, sale
	*Citrus medica* var. *sarcodactylis*	1	0	Fruit	Few households and little land area per household	Perennial, more fragrant, human consumption, sale
	*Citrus reticulata*	1	0	Fruit	Some households and little land area per household	Perennial, good tasty, sweet, human consumption, sale
Solanaceae	*Capsicum annuum*	2	0	Spice	Many households and little land area per household	Low and middle yield, spicy, human consumption
	*Capsicum frutescens*	1	0	Spice	Many households and little land area per household	Low and middle yield, spicy, human consumption
	*Nicotiana tabacum*	0	2	Cash	No households	Low yield, sale
	*Solanum betaceum*	1	0	Vegetable	A few households and little land area per household	Perennial, human consumption, sale
	*Solanum etuberosum*	2	0	Vegetable	A few households and middle land area per household	Middle and high yield, human consumption, sale, fodder
	*Solanum lycopersicum*	1	1	Vegetable	For local variety, some households and little land area per household	For local variety, low yield, good tasty, human consumption, sale
					For imported variety, some households and a large land area per household	For imported variety, high yield, human consumption, sale
	*Solanum melongena*	1	0	Vegetable	Many households and little land area per household	Middle yield, human consumption
Theaceae	*Camellia sinensis*	3	0	Cash	A lot of households and large land area per household	Perennial, good smell, human consumption, sale
Zingiberaceae	*Amomum villosum*	0	1	Cash	A lot households and large land area per household	Perennial, growing in national forest, consumption, sale
	*Curcuma longa*	1	0	Spice	Some households and little land area per household	Perennial, yellow root, human consumption, sale
	*Zingiber officinale*	2	0	Spice	A lot of households and middle land area per household	Perennial, yellow root, human consumption, sale

Corn and rice were the main food crops, and were utilized both for human consumption and livestock fodder. Corn included 10 local varieties and 13 new varieties and was mainly used for livestock fodder grain (66.3%), followed by alcohol production (25.4%) and human consumption (7.1%), and was rarely for market sale (1.2%) (Table 3). Local villagers primarily consume corn made into an alcoholic drink, but do consume some fresh corn directly. In the region, there were 7 local varieties and 16 new imported varieties of paddy rice and 21 local varieties and no new imported varieties for upland rice. Almost all rice (90.6%) grown by local farmers was used for household consumption. Only a small amount of rice was used for alcohol production (5.8%) and market sale (3.6%) (Table 3).

**Table 3:** Function of different crops in Xishuangbanna

Crop type	Eating (%)	Making alcohol (%)	Sale (%)	Fodder (%)	Others (%)
Maize	7.1	25.4	1.2	66.3	0
Rice	90.6	5.8	3.6	0	0
Vegetable	42.5	0	7.1	50.4	0
Spice	97.5	0	2.5	0	0
Cash	21.6	0	78.4	0	0
Oil crop	92.5	0	7.5	0	0
Fruit	60.4	0	39.6	0	0
Cloth	0	0	0	0	100

Vegetables grown included 68 local varieties and 15 new imported varieties, which were mainly used for livestock fodder (50.4%) and household consumption (42.5%), and rarely for market sale. Spices grown included 22 local varieties and 2 new imported varieties, of which most were consumed by households (97.5%) and little for market sale (2.5%). The six oil crops (92.5%) and one cloth crop (100%) were mainly grown for household consumption. Fruit crops were grown for human consumption (60.4%) and market sales (39.6%). Most of the cash crops were introduced by local government and mainly grown for cash sales (78.4%) with a small amount grown for human consumption (21.6%) (Table 3).

#### Livestock genetic resources and functions

There were 15 livestock varieties, belonging to six major species in Xishuangbanna (Table 4). Animals raised included cattle, goats, pigs, chickens, ducks, and geese. Most cattle raised were yellow cattle and buffalo, both of which were local varieties. Yellow cattle were mainly used for transporting goods, including maize, rice and fodder collected in swidden land, and buffalo mostly used for plowing. The other uses of yellow cattle and buffalo included providing manure, labour, household consumption, and cash (Table 5). Cattle were never used to produce milk and rarely killed for household consumption, rather they tended to be consumed at big events such as funerals, wedding and religious activities in the village. Cattle manure was essential to maintain good agricultural yields. Usually, cattle served as a store of value (a valuable disposable asset) and were rarely sold, and when they were sold, it was not usually a planned transaction. Because of the high labour requirements of raising cattle, they were often owned and raised jointly by many households in wastelands and mountains. Goats provided manure, were sold or saved as store of value, and their meat was consumed at home. Goat faeces were highly valued as a fertilizer, and were said to have a better effect than pig or cattle manure. Goats required a household member to herd them full-time because they run quickly.

**Table 4:** Summary of type and characteristics of livestock in Xishuangbanna

Name	Origin	Population	Feed	Uses and function	Other characteristics
Yellow cattle	Local	Reduced	No stall-raising	Transport, manure, meat, sale, exchange, store of value	Various appearance colors, small size, no produce milk
Buffalo	Local	Reduced	No stall-raising	Plowing, manure, meat, sale, exchange, store of value	Black and white colors, big size, no produce milk, plowing before and now instead of plowing machine, many household raised jointly
Goat	Local	Reduced	No stall-raising	Consumption, manure, meat, sale, store of value	Various appearance colors, small size, fast running
Donggua pig	Local	Reduced in low elevation, increased in middle and high elevation	No stall-raising before and pen-raising now	Consumption, manure, meat, lard, gifts, exchange, savings, sale	Small size and small ear, slow growth, more 1 year raising, 50-70 kg, high price, high nutritional value, good tasty, more fodder grain, few diseases
Small ear pig	Local	Reduced in low elevation, increased in middle and high elevation	No stall-raising before and pen-raising now	Consumption, manure, meat, lard, gifts, exchange, savings, sale	Small size and small ear, slow growth, more 1-2 year raising, 70-80 kg, high price, high nutritional value, good tasty, more fodder grain, few diseases
Landrace pig	Imported	Reduced	No stall-raising before and pen-raising now	Consumption, manure, meat, lard, savings, sale	Big size and big ear, fast growth, 3-5 months raising, 150-200 kg, low price, fattier, low nutritional value, low tasty, few disease
Duroc pig	Imported	Steady	No stall-raising before and pen-raising now	Consumption, manure, meat, lard, savings, sale	Big size and big ear, fast growth, 3-5 months raising, 150-200 kg, low price, fattier, low nutritional value, low tasty, few diseases
Shuanghui pig	Imported	Reduced	No stall-raising before and pen-raising now	Consumption, manure, meat, lard, savings, sale	Big size and big ear, fast growth, 3-5 months raising, 150-200 kg, low price, fattier, low nutritional value, low tasty, few diseases
Chahua chicken	Local	Steady	No stall-raising	Consumption, sale, exchange, gifts, meat, eggs	Small size and small feet, slow growth, 1 year raising, 1 kg, high price, high nutritional value, high tasty, more hard bones, few diseases
Cultivated chicken	Local	Increased	No stall-raising	Consumption, sale, exchange, gifts, meat, eggs	Medium size, fast growth, 6 months raising, 1-3 kg, high price, more eggs, high nutritional value, high tasty, few diseases
Fighting chicken	Imported	Reduced	No stall-raising	Cockfighting game	Long feet, cockfighting game on Thursday and Friday
Waidi chicken	Imported	Reduced	No stall-raising	Consumption, sale, exchange, gifts, meat, eggs	Big size, fast growth, 3-5 months raising, 2-5 kg, low price, low tasty, fattier
Drought duck	Local	Reduced	No stall-raising	Consumption, sale, exchange, gifts, meat, eggs	Small size, low growth, 6 months raising, 2-4 kg, high price, good tasty
Water duck	Local	Reduced	No stall-raising	Consumption, sale, exchange, gifts, meat, eggs	Big size, fast growth, 3-5 months, 3-4 kg, low price, low tasty
Goose	Imported	Reduced	No stall-raising	Consumption, sale, exchange, gifts, meat	Big size, fast growth, 6-7 months raising, 5-6 kg, medium tasty

**Table 5:** Purposes of livestock raising by villagers in Xisuhangbanna

Type	Cattle		Buffalo		Swine		Goat		Poultry	
	F	%	F	%	F	%	F	%	F	%
Sale	6	4.9	5	3.1	45	12.7	34	47.9	94	26.1
Bank	32	26	47	29.6	77	21.6	13	18.3	35	9.7
Consumption	15	12.2	12	7.5	135	37.9	13	18.3	198	55
Plowing	0	0	20	12.6	0	0	0	0	0	0
Transportation	13	10.6	0	0	0	0	0	0	0	0
Manure	55	44.7	75	47.2	78	21.9	7	9.9	0	0
Exchange	2	1.6	0	0	21	5.9	4	5.6	33	9.2
Total	123	100	159	100	356	100	71	100	360	100

*F* Frequency of respondents, multiple responses

Pigs raised included two local varieties (Donggua pig and small ear pig) and three new imported varieties (Landrace pig, Durloc pig and Shuanghui pig). Generally, new imported pigs grown faster, were more easily raised, and reduced more fodder grain than traditional varieties. However, they were less flavorful and had lower nutrient levels and fetched a lower market price. A local pig took about at least one year to reach consumable size, but a newly import pig just required 3-6 months. The primary function of pigs was for household consumption. Pigs were also slaughtered for funerals, weddings, house building, rituals, and religious activities. When pen-raised, pig manure contributed to the compost that was put on corn and vegetable fields. Pigs are rarely sold and piglets were given away to friends and relatives as gifts.

Chickens were raised by almost every household and numbers changed rapidly because a household may raise many large and small chickens at any one time. Chickens raised includes two local varieties (Chahua chicken and cultivated chicken) and two new imported varieties (Fighting chicken and Waidi chicken). Compared to traditional varieties, new imported chickens usually grew faster, were more easily raised, fattier, and heavier. However, they were less flavorful and had lower nutrient content and fetched a lower market price. A chicken took about six months to reach a saleable and consumable size. Chickens were easily sold in the village and can be converted quickly into small amounts of cash to be used for buying daily necessities. In addition to cash sales, chickens can be exchanged for wine, grain, piglets, and other items. They were also frequently given as gifts between relatives and friends. Fighting chickens originated from Myanmar and Thailand and were mainly used for cockfighting. A small number of households also raised ducks and geese for household consumption, exchange and rarely sale.

### Agrobiodiversity changes

#### Changes of crop diversity

According to field surveys, agricultural farming systems and crop diversity in Xishuangbanna have changed greatly. In order to support livelihood development and ecological protection of ethnic minority areas in Yunnan, national and provincial governments have issued a series of policies, such as poverty alleviation, forest conversion and agricultural technology extension. In 2002, the Sloping Land Conversion Program (SLCP) was implemented in Xishuangbanna. Apart from retaining paddy rice fields, permanent cultivation fields and vegetable gardens, almost all rotational fields (swidden) and some arable land were incorporated in the conversion program. Villagers who took part in the program would be provided with grain subsidies or cash for five to eight years. However, they were required to plant trees and some cash crops and ecological crops (rubber, tea and bamboo) as the swidden lands were converted. The implementation of the SLCP has achieved great success in economic, environment and ecological terms. Household income from cash subsidies and from cultivation of cash crops in converted swidden lands was quickly increased. Land degradation was reduced, natural and artificial vegetative cover was increased and soil and water loss was reduced. However, this program brought traditional cultivation to an end, threatened survival and continuity of crop species diversity and traditional culture in Xishuangbanna. Since the implementation of the SLCP, agricultural land use and diversity of crop varieties have decreased. In 2002, the cultivated area under swidden agriculture accounted for 58% of all cropland, but now swidden cultivation is almost completely eliminated. Traditional crop varieties, especially some upland rice and sticky corn have been lost. Many traditional crops disappeared because the yields were lower when planted in permanent fields rather than in swiddens. Moreover, as household income from cash subsidies and from cultivation of cash crops in converted swidden lands improved livelihoods, food crops were neglected by local villagers, especially cultivation and conservation of old varieties.

Every year, local agricultural technology service sectors would introduce different new crop varieties and technology in Xishuangbanna. At the beginning of implementation, to encourage local villagers to grow these species, local governments adopted a series of favorable incentives, such as providing free seed, chemical fertilizers, herbicides, and plastic sheeting. New crops had generally higher yield and faster growth than local crops and new technologies were more convenient and reduced labour, so these new crops were easily accepted and used by villagers. The main new crop varieties were maize, rice and vegetables, and new technologies were comprised of plastic sheeting, auxin, herbicide, and pesticides. With these promotions, local agricultural production has become more and more dependent on these technologies.

Many villagers reflected that new varieties were usually higher yield but were less flavorful, of lower nutritional value, preferred more fertile soil, and less resistant to diseases compared to traditional varieties. Some traditional crops, such as sticky maize and sticky rice, play an essential role in local traditional rituals such as traditional festivals, ancestor worship and religious activities. Therefore, in addition to planting a large area of new corn varieties to feed livestock, some villagers also devoted a small area to old varieties for household consumption and rituals. In low altitude areas, due to large areas in rubber plantation, local villagers only had a small areas available to plant new rice and corn varieties and a few new vegetables. Middle altitude areas were very suitable for planting rubber and banana, and high altitudes were only suitable for growing tea, sugarcane, walnut, and pine. In these areas, local villagers had more lands to plant more crops, and some traditional varieties were only suitable for planting at high altitude. As market prices increased and more and more people preferred traditional food and diets, some modern commercial companies and others have rented or bought arable land at low and middle altitudes to grow traditional maize, vegetables and spices.

#### Changes in livestock diversity

The number and variety of livestock resources have greatly declined in Xishuangbanna. Some livestock used in transport such as horses and mules were no longer employed by local communities. In order to improve local livestock breeds and promote local animal husbandry production, some new livestock breeds, especially pigs and chickens were often introduced by local governments. Hybridization of either pigs or chickens has resulted in the erosion of local varieties. Most yellow cattle, buffalo and goats were still from traditional varieties, but their populations were in decline. This was because many herding places have gradually been replaced by a large area plantation of economic crops such as tea, rubber, bamboo, and banana. Moreover, with a lack of special grasslands, villagers had no incentive to put more labour and material resources to herd livestock. Currently, there were very few cattle and goats raised in low altitude areas. Although there were some villagers raising a large number of cattle and goats at high altitudes, genetic diversity of cattle decreased as population decreased, as did the genetic diversity of goats. Overall, the number of pigs showed an upward trend because most pigs were used for household consumption and rarely for market sale. Local villagers would consider nutritional value and taste of pigs when they selected variety. Compared to new varieties, local traditional pigs had faster growth, better taste and were less fatty, so local villagers tended to raise old varieties. Moreover, due to high market price, preference by consumers and strong support from local government, traditional pig varieties such as Donggua pigs and small ear pigs were increasingly raised at middle and high altitudes.

The population of chickens was steady and/or slightly increased at middle and high altitudes, especially for Chahua chicken and cultivated chicken. Compared to traditional varieties, new imported chickens could usually grow faster, were more easily raised, fattier, and heavier. However, they were less flavorful and had lower nutrient levels and fetched a lower market price. Therefore, local villagers preferred to raise traditional varieties of chickens and a small number of new varieties. The number of local ducks and new imported geese were significantly decreased.

#### In situ conservation of agrobiodiversity

##### Crops conservation

Currently, the protection of crop diversity has not received enough attention by relevant interest groups in Xishuangbanna. Most of crops were usually used as the food supply in many communities. Some crops (such as sticky rice and sticky corn) had important significance for traditional rituals, but were rarely used for market sale, so many villagers did not think they had special protection value. Villagers and communities consciously or unconsciously protected crops through two ways, via preserved seeds or via planted crops. The main method of storing crop seeds was to put them on the fireplace in a residence. This method served to dry crop seeds and prevent insect damage. In addition, most families had an independent grain house to preserve crop seeds. After returning swidden to forest, some villagers still kept crop seeds but did not continue to plant them. Villagers thought that many crop seeds could only be stored for 1-3 years, but some may be preserved for 8 years.

The survey found that a lot of villagers planted a small land area or some villagers grew a large area of traditional crop varieties. In low altitudes, villagers usually planted a large area of new varieties of crops and very small land traditional crop varieties. However, if communities were close to market or had access to vehicles, villagers might plant a large area of old varieties, especially traditional vegetables for market sale. At high altitudes, many villagers mainly planted new varieties, but because more land was available, they also planted old varieties on some of the available land. While rubber and banana crops were at relatively young stages, villagers tended to grow old varieties such as upland rice, corn and vegetable simultaneously on the plantations.

#### Livestock conservation

Villagers kept livestock through breeding of traditional varieties. Livestock such as yellow cattle, buffalo and goats were all local traditional breeds. Before the SLCP, these livestock were not stall-raised and were freely grazed by individual family, but now due to increased land in cash crops, many households grazed jointly. At low altitudes, only a small number of villagers raised a few cattle and goats, but at high altitudes a small number of villagers raised larger numbers. Pigs were freely grazed before the SLCP and now were pen-raised in pigsties. Local villagers preferred traditional pork, so at middle and high altitudes, most families raised several Donggua pigs and small ear pigs. In some communities, some villagers established special places to breed old varieties for market sale. Chickens were the main source of meat raised by villagers. Local villagers preferred to breed traditional chicken varieties such as Chahua chicken and cultivated chicken. Almost all families raised ducks before the SLCP, but subsequently only a small number of villagers raised a large number of ducks and geese for sale.

## Discussion and recommendations

For many agricultural people inhabited in upland mountainous regions, swidden agriculture is an integral part of not only natural-resources management and genetic-resources conservation, but also of ethnic identity and biocultural heritage [6, 7]. In areas rich with natural biodiversity, the persistence of swidden cultivation is mostly seen as a ‘problem’ that obstructs the achievement of conservation objectives, and policies originating from a forest-conservation perspective often seek to eradicate swidden agriculture. However, traditional rotational shifting cultivation contributes to the maintenance of diversity of both crop and animal genetic resources, and these play important roles in maintaining cultural identity [6].

Xishuangbanna is one of the ethnic minority areas in Yunnan Province and swidden agriculture is central to agrobiodiversity, livelihoods and traditional culture there [7]. Because of the location of Xishuangbanna neighboring Laos, Myanmar and Vietnam, some crop varieties are likely to be unique to the region. Some studies reported that there were more than 577 cultivated plants in Xishuangbanna before 2011 and most of them were related to swidden agricultural practice [7, 11, 12, 19, 20]. There were over 100 varieties of upland rice in swidden fields among just a few Hani and Jinuo communities in Xishuangbanna before 1996 [7]. Genetic diversity, variety diversity and species diversity of agricultural resources in Xishuangbanna have declined and some crop varieties have even disappeared. First of all, land in agricultural production was decreased greatly because most swidden land was replaced by rubber, tea, banana, coffee, bamboo, and pine. Secondly, plantation style crops tend to towards monocultures and most food crops are new varieties. Finally, local traditional crop varieties, area over which they are planted, and the number of villagers cultivating them are gradually reduced.

For any ethnic group engaged in agriculture, agricultural production is a core part of the ethnic culture [6]. In Xishuangbanna, traditional agriculture is an important part culture feature for ethnic minorities such as Dai, Hani, Yi, Yao, Bulang, and Lahu. Traditional agriculture, food culture, other cultural aspects, traditional ecological knowledge, religion, and the concept of the universe are highly interrelated [12]. Along with implementation of the SLCP and livelihood development, many younger villagers lost interest in traditional culture, and were unwilling to return to traditional practices for agricultural production.

Besides in situ conservation, through sustainable uses, including techniques developed for propagation, cultivation, on farm and off farm management, new variety breeding, and scientific studies are also important protection measures for agrobiodiversity [21]. In many mountain areas, local food security is mostly dependent on conservation of agricultural biological resources and plant resources, so in situ conservation measures by local farmers are very important [22]. Stemming from our discussions with local villagers and relevant government agencies, we recommend the following conservation measures:

1. Promote consensus among different stakeholders on the value of agrobiodiversity conservation: This research found that new imported varieties of crops and livestock were widely accepted by local villagers, technicians and officials but there needs to be a way to alleviate the contradiction between traditional variety conservation and new variety extension. Many personnel promoting agricultural technology and many villagers have not recognized the importance of traditional agricultural biological resources, so it is necessary to implement a series of exchange meetings and other communication to promote consensus among different stakeholders on the value of agrobiodiversity conservation.
2. Encourage households to conserve traditional varieties in permanent plots: The advantages of traditional variety conservation through individual household cultivation may be realized by ensuring local villagers can take personal ownership of germplasm resources and publically participate in biodiversity conservation promotion [6]. However, some traditional low yielding varieties are not protected effectively, so it may be more feasible to utilize collective community farmland to conserve traditional crops in these cases. At the local farming system level, each household could contribute to land, seeds and the labour force. Each village could decide on participation, seed and labour contributions and how to distribute outputs.
3. Convene seed exchange fairs among farmers: Crop diversity can be enhanced by facilitating seed exchange among farmers [23]. Many households believe that other farmers are retaining traditional-crop seeds and expect that if, in the future, they want to plant these crops, they will be able to obtain seeds through seed exchanges [6, 24, 25]. In fact, information on traditional crop conservation is rarely exchanged among different communities. In order to raise awareness of traditional crop conservation for villagers and local township government, some traditional cultural-exchange meetings should be convened in different communities. During seed fairs, villagers could bring traditional seed varieties and exchange knowledge about seeds, as well as exchanging the germplasm itself. The fairs could also provide an opportunity for local government officials to understand the range and characteristics of traditional seed varieties and their current conservation status. Meanwhile, in order to improve farmers’ awareness and encourage them to protect older varieties, special competitions and prizes should be designated in various categories, such as traditional crop-variety preservation, traditional food making, song and dance, and traditional costume making [6].
4. Make a visual documentary of the indigenous knowledge related to cultivation: Considering the cultural significance and abundance of traditional knowledge of various traditional resources, it is necessary to record the status, distribution, use and management, and cultural values of some agricultural biological resources, by using a digital video camera [6]. When the video is completed, an ethnic language version can be used to educate school children about traditional agriculture and related culture. Chinese language version may also be useful to support dialogue with policymakers in the future. The video documentary format is conducive to improve understanding of environment and minority culture for the younger generation. Moreover, local traditional knowledge is also recorded and preserved with this method.
5. Provide traditional agricultural products to tourists: The feasibility of developing processed products using traditional crop varieties for sale to tourists can be explored as a means to promote agrobiodiversity conservation [6]. Local government agencies could help villagers and communities to establish some tourism centres. These centres can organize local villagers to grow traditional crops, raise traditional livestock, collect wild medicines and cash plants, and make traditional costumes for markets. Traditional seeds, crop products, livestock products, traditional handicrafts, and traditional cultural shows would help educate and entertain tourists. This is regarded as a very important strategy for strengthening the linkage among agrobiodiversity conservation, preservation of traditional culture and livelihood development [6].
6. Ex situ conservation of agrobiodiversity: For rare and endangered agricultural crop and animal species in ethnic minority regions, ex situ conservation is another feasible strategy for agrobiodiversity conservation and may be particularly appropriate for certain crop or livestock varieties. Ex situ conservation could include seed banks/germplasm banks, zoos and botanical gardens [21]. In addition to preserving endangered species, ex situ conservation also provides good sources of crop and livestock materials for research as well as providing additional sources of these materials for famers if needed.

## Conclusion

Xishuangbanna is a multinational region considered to be a global biodiversity and cultural hotspot. The study found that there are abundant farming crop and livestock resources and that agrobiodiversity is central to local livelihood and traditional culture in Xishuangbanna. However, due to rapid economic development and land use changes, especially under implementation of the SLCP since 2002, local agrobiodiversity and related traditional culture have suffered losses and faced tremendous challenges. Some traditional crop and livestock resources have declined and some have even disappeared. In situ conservation measures such as preserved seeds, planted crops and livestock are very important methods for conserving local agrobiodiversity but need to be enhanced in Xishuangbanna. Thus in future conservation of agrobiodiversity, sustainable protection measures based in local communities should be considered and adopted.
